# New perspectives on mobile phone addiction based on network analysis

**DOI:** 10.3389/fpsyt.2025.1665673

**Published:** 2025-10-13

**Authors:** Rui Qiu, Zhihua Guo, Xianyang Wang, Yushan Li, Mengze Li, Zhu Xia

**Affiliations:** Department of Military Medical Psychology, Air Force Medical University, Xi ‘an, China

**Keywords:** mobile phone addiction, network comparison test, central node, network analysis, effortful control

## Abstract

**Background:**

Mobile phone addiction represents a widespread concern associated with negative health consequences, influenced by various protective and risk factors. Nonetheless, two significant gaps persist in the literature: the examination of gender differences in the interplay among mobile phone addiction symptoms, and the analysis of dimension-level associations between mobile phone addiction and its determinants. These gaps motivated the current investigation. This study aimed to elucidate the interconnections among symptoms of mobile phone addiction through the development of a relational network and the identification of key central symptoms. Furthermore, it investigated the influence of fundamental psychosocial factors on mobile phone addiction, with a specific focus on gender-related differences.

**Methods:**

The sample comprised 1,684 adults. Participants completed validated self-report instruments, including the Mobile Phone Addiction Tendency Scale (MPATS) and the Connor-Davidson Resilience Scale (CD-RISC), to assess mobile phone addiction and relevant psychosocial factors. Network analysis techniques were employed to construct two models: a standalone symptom network of mobile phone addiction and an integrated network incorporating addiction symptoms and influencing psychosocial factors. Additionally, gender-stratified analyses were conducted to compare network structures and centrality metrics between male and female groups.

**Results:**

Across both genders, the most robust associations within the mobile phone addiction symptom network were confined to items within the same MPATS dimension. The strongest edge common to both networks connected Item 4 (“I would feel bad if I did not use my phone for a long time”) and Item 6 (“I would feel lonely without my mobile phone”), both reflecting withdrawal symptoms. The second strongest association linked Item 7 (“I feel more confident when I communicate with others using my phone”) and Item 16 (“I feel more comfortable when I communicate with others via cell phone”), which pertain to the social comfort dimension. Regarding central symptoms, Item 15 (“In class/at work, I often take the initiative to focus on my mobile phone, which affects the lesson/work”) emerged as the central node among males, whereas Item 6 (“I would feel lonely without my mobile phone”) was central among females. Within the integrated network, the strength dimension of psychological resilience served as the central node for both genders. Additionally, in the male sample, the positive reappraisal dimension of cognitive emotion regulation strategies was identified as the central node, whereas the catastrophizing dimension emerged as the central node in the female sample.

**Conclusion:**

The results demonstrate significant gender disparities within both the isolated mobile phone addiction symptom network and the comprehensive network that includes psychosocial variables. Strong connections were observed particularly within the withdrawal and social comfort domains, accompanied by gender-specific central symptoms—namely, task-interfering phone use among males and feelings of loneliness among females. These findings contribute to a deeper comprehension of the psychopathological processes associated with mobile phone addiction. Moreover, they identify specific focal points for the formulation of gender-responsive intervention strategies designed to reduce mobile phone addiction.

## 1 Introduction

Due to the progress and development of science and society, smartphones, which facilitate communication, entertainment, education, and other functions, play an increasingly important role in people’s lives (1). However, as smartphones become more widely used, frequently used, and used for longer periods, the emerging smartphone use habits can increase the risk of mobile phone addiction; accordingly, many people now exhibit mobile phone addiction, which seriously affects their work and lives and poses tremendous societal challenges (2). Mobile phone addicts usually exhibit signs of relapse, withdrawal symptoms, salience, and impulsivity, which take the form of impaired social functioning due to their uncontrollable overuse of smartphones (3).

With the growing concern surrounding mobile phone addiction, researchers have extensively examined the associated risk factors and consequences, developing various theoretical frameworks to elucidate the determinants of mobile phone addiction from perspectives such as psychological needs and external pressures. Notably, among these frameworks, the Interaction of Person-Affect-Cognition-Execution (I-PACE) model has garnered significant attention and validation within the academic community.

The I-PACE model integrates multiple factors that influence mobile phone addiction and can account for the causes of mobile phone addiction effectively (4). On this basis, the I-PACE model is employed in this study, which incorporates individual psychological resilience (5), general future time perspective (6), and the need for uniqueness (7) as personal characteristic factors as well as emotion regulation (8) as an affective state factor, cognitive emotion regulation strategies (9) as a cognitive ability factor, and effortful control (10) and mind wandering as executive function factors. The aim of this study is to incorporate the factors influencing mobile phone addiction into this model in a more comprehensive way.

Psychological resilience is defined as the capacity to sustain or restore psychological well-being in the face of adversity (5). Psychological resilience is defined as the capacity to sustain or restore psychological well-being in the face of adversity. Consequently, these individuals are more likely to preserve psychological health during challenging circumstances and exhibit a decreased propensity for violent behavior, substance addiction, and other maladaptive behaviors (11–13).

For instance, when confronted with academic setbacks, resilient individuals are more likely to modify their study plans instead of engaging in prolonged social media use, thereby mitigating the likelihood of developing addictive mobile phone behaviors.

General future time perspective denotes an individual’s overarching psychological capacity to anticipate, conceptualize, and shape the future, which is primarily manifested through cognitive, affective, and behavioral orientations toward self-development (6); This perspective serves to mitigate mobile phone addiction by facilitating the prioritization of long-term goals (14, 15). Individuals possessing a strong future time perspective are inclined to perceive excessive mobile phone use as an impediment to the attainment of long-term objectives, such as career progression or academic success, and consequently engage in proactive regulation of non-essential phone activities, for instance, refraining from gaming during work hours. Such regulation diminishes the incidence of impulsive or compulsive phone use, thereby reducing the risk of addiction (16).

The need for uniqueness represents an inherent psychological drive motivating individuals to seek differentiation from others, a fundamentally adaptive trait when manifested in moderation, such as through personalized hobbies or values that reinforce self-identity. However, when this need becomes excessive, smartphones serve as an accessible and low-cost platform to fulfill intensified desires for distinctiveness. Individuals may engage in oversharing carefully curated “unique” life experiences on social media, utilize specialized applications to distinguish themselves from peers, or cultivate a distinctive online persona through compulsive interactions with their devices. This dependence on smartphones to validate uniqueness initiates a self-reinforcing cycle: increased device usage aimed at achieving distinctiveness heightens reliance on online feedback to affirm one’s sense of individuality, which in turn perpetuates compulsive use and ultimately contributes to mobile phone addiction (7).

Emotion regulation and cognitive emotion regulation strategies constitute interconnected facets of adaptive psychological functioning; however, impairments in these processes contribute to mobile phone addiction via distinct pathways. Emotion regulation refers to the capacity to modulate emotional states through deliberate strategies, such as reframing situational appraisals, to align with environmental demands. Effective emotion regulation reduces negative affective states, including anxiety and loneliness, without resorting to external distractions (8).

Cognitive emotion regulation strategies, specifically the mental processes employed to interpret and manage emotional information, further refine this capacity. Adaptive strategies, such as positive reappraisal and refocusing on planning, promote psychological well-being and prosocial development, thereby decreasing the propensity to use smartphones as an escape from distress. Conversely, maladaptive strategies, including rumination and catastrophizing, exacerbate negative emotions and drive individuals to rely on smartphones as a rapid means of alleviating emotional discomfort, reinforcing addictive behaviors over time (9).

Effortful control, a fundamental aspect of self-regulation, denotes the ability to inhibit dominant impulsive responses and engage in goal-directed behaviors, such as resisting the urge to check one’s phone during work to maintain task prioritization. This capacity is closely associated with individual adaptability; those exhibiting strong effortful control demonstrate enhanced adaptive functioning by effectively managing stressors and sustaining focus without dependence on smartphones. In contrast, deficient effortful control compromises adaptability by favoring immediate gratification, such as responding to phone notifications, over long-term objectives, resulting in frequent impulsive phone use. Over time, this impulsivity evolves into compulsive behavior, thereby increasing the risk of mobile phone addiction (10).

Mind wandering, defined as the involuntary diversion of attention toward unrelated thoughts, imaginative scenarios, or past experiences during periods of idleness or task engagement, contributes to mobile phone addiction by undermining attentional control (17).

Frequent episodes of mind wandering impair sustained concentration on ongoing activities, such as reading or working, creating attentional gaps that individuals often fill with unplanned phone use, for example, automatically reaching for their device when their mind drifts. Repeated engagement in this compensatory “attention-filling” phone use gradually consolidates into habitual and eventually addictive behavioral patterns (18). Building upon the aforementioned mechanisms, the current study posits that protective factors—such as resilience, a general future time perspective, emotion regulation, adaptive cognitive emotion regulation strategies, and effortful control—will be inversely associated with mobile phone addiction. Conversely, risk factors—including an excessive need for uniqueness and frequent mind wandering—are expected to demonstrate positive associations with mobile phone addiction. Furthermore, maladaptive cognitive emotion regulation strategies, as a subset of cognitive emotion regulation, are hypothesized to be positively correlated with mobile phone addiction, reflecting their contributory role in exacerbating distress-driven mobile phone use. Gender influences mobile phone dependency and preferences regarding internet usage, in which context men tend to view mobile phones as tool-like devices, while women use mobile phones as a medium to develop and maintain social contacts (19). Men are more proactive with regard to seeking a sense of power and fulfilling their sexual fantasies, whereas women prefer to remain anonymous online, thereby concealing their true identities and using the internet to seek intimate friendships and comfort (20). Mok and colleagues employed latent class analysis techniques in their study, which revealed that female college students are more prone to exhibit smartphone addiction than are male college students; furthermore, as dependency on smartphones increases, the individual’s anxiety index and neurotic traits also increase (21).

Improper mobile phone use can give rise to a variety of physiological and psychological discomforts in individuals, which can be divided into four categories: negative reactions on a physiological or psychological level when the individual is not engaged in mobile activities, the central state of mind and behavior associated with mobile phone use, the role of mobile phone usage in interpersonal communication, and a series of negative emotional changes resulting from mobile phones (22). These dimensions constitute the core indicators of and factors associated with mobile phone addiction disorder. The construction of a mobile phone addiction network model can also expand the extant research on issues related to addiction.

The studies referenced above demonstrated that the problem of addiction is not caused by a single factor but rather involves complex interactions among brain physiology, behavioral symptoms, the proximal environment, and distal culture (23). As a psychological disorder, mobile phone addiction can be viewed essentially as a complex and variable network system featuring feedback loops among various factors. Individuals are thus integrated into a dynamic self-organizing system, which makes it difficult to address the symptoms of mobile phone addiction disorder (24). Previous researchers have used network theory to study various psychological disorders and have developed corresponding various network analysis models (25). For example, network analysis models have been developed to investigate posttraumatic stress disorder, anxiety disorders, mood disorders, and personality disorders (26). Investigating the issue of mobile phone addiction from a dynamic system perspective can help us construct a complete risk factor model for mobile phone addiction, provide us with a framework for integrating factors at multiple levels, and help us analyze the effects of different factors on mobile phone addiction (27).

This study pursues two primary objectives: firstly, to delineate the interrelationships among symptoms of mobile phone addiction by mapping their connections and identifying the central, driving symptoms; secondly, to investigate the influence of psychosocial factors, namely resilience, cognitive emotion regulation, and effortful control. Additionally, gender-stratified analyses were conducted with two principal aims: to reveal gender-specific connectivity patterns within both the addiction symptom network and the psychosocial factor network, thereby enabling the distinct identification of core driving symptoms and key influences for each gender; and to establish a gender-sensitive empirical basis for the development of targeted interventions. By integrating gender-stratified analyses, this research provides a more nuanced and comprehensive understanding of mobile phone addiction.

## 2 Methods

### 2.1 Participants and procedures

The questionnaire used to support this study was created using the Wenjuanxing platform (https://www.wjx.cn/) in March 2023, and the questionnaires were distributed and collected via various social media platforms. A total of 1932 healthy adults participated in this study. After the exclusion of invalid questionnaires, the sample featured 1684 responses in total, including 1383 males and 301 females. Approximately 21.6% of the participants were under 20 years old, and 57.4% of the participants were between the ages of 21 to 30 years. To ensure the quality of the collected questionnaires, we used the following three criteria to exclude invalid questionnaires: ① questionnaires featuring response times of less than 900 s or more than 1800 s; ② responses featuring incorrect answers to the lie detector question;③ questionnaires on which the same option was chosen for more than 90% of the questions included in the same scale. After statistical screening, 1,684 valid samples were retained, for a validity rate of 87.16%. All the data were collected in March 2023. The participants represented 30 provincial administrative regions throughout China. The demographic information of the participants is shown in **Table 1**.

**Table 1:** Demographic characteristics of the research participants.

Sample characteristics		Frequency	Percentage (%)	Cumulative percentage (%)
Gender	Male	1,383	82.1	82.1
	Female	301	17.9	100.0
Only child status	Only child	546	32.4	32.4
	Not only child	1,138	67.6	100.0
Age	≤ 20 years old	364	21.6	21.6
	21–30 years old	966	57.4	79.0
	31–40 years old	211	12.5	91.5
	41–50 years old	76	4.5	96.0
	> 51 years old	67	4.0	100.0
Educational background	Junior middle school or below	50	3.0	3.0
	Senior high school or junior college	878	52.2	55.2
	Undergraduate	735	43.6	98.8
	Postgraduate education or higher	21	1.2	100.0

### 2.2 Measures

#### 2.2.1 The mobile phone addiction tendency scale

The MPATS is a self-reported instrument that include 16 items used to assess subjects’ tendencies toward mobile phone addiction (22). The scale features four dimensions, namely, withdrawal symptoms; salience; social comfort and mood changes. All items are scored on a 5-point Likert scale ranging from 1 (completely inconsistent) to 5 (completely consistent). Higher scores indicate higher levels of mobile phone addiction. In the current study, the Cronbach’s alpha coefficients were 0.87 for withdrawal symptoms, 0.85 for salience, 0.82 for social comfort, 0.76 for mood changes and 0.94 for the total scores.

#### 2.2.2 The cognitive emotion regulation questionnaire

The CERQ focuses on both adaptive and maladaptive cognitive strategies, which define an individual’s characteristic approach to the task of managing stress-related events (9). The Cognitive Emotion Regulation Questionnaire (CERQ) used in this study is the Chinese version developed by Zhu Xiongzhao et al. and validated through psychometric testing (28). The adaptive cognitive strategies scale includes five subscales: acceptance, refocus on planning, positive refocusing, positive reappraisal and putting into perspective. The maladaptive cognitive strategies scale includes four subscales: self-blame, blaming others, rumination or focus on thought and catastrophizing. Each subscale consists of 4 items, which are scored on a 5-point scale ranging from 1 (almost never) to 5 (almost always). In the current study, Cronbach’s alpha coefficients ranged from 0.75 (self-blame) to 0.94 (refocus on planning).

#### 2.2.3 The general future time perspective questionnaire

The GFTPQ is a 20-item self-report scale used to measure individuals’ future time perspectives (29). It includes five factors: future efficacy, purposive consciousness, far-reaching goal orientation, future image and behavioral commitment. Each item is scored on a 4-point scale ranging from 1 (completely consistent) to 4 (completely inconsistent). In the current study, the Cronbach’s alpha coefficients ranged from 0.62 (future image) to 0.89 (behavioral commitment).

#### 2.2.4 The emotion regulation questionnaire

The ERQ is a 10-item self-report scale used to measure emotion regulation strategies (8). This questionnaire includes two factors: reappraisal and suppression. Emotion reappraisal is defined as an antecedent-focused strategy in which a person attempts to change how they think about a situation with the goal of changing its emotional impact. Emotion suppression is defined as a response-focused strategy in which a person attempts to inhibit the behavioral expression of their emotions. Each item is scored on a 7-point Likert scale ranging from 1 (strongly disagree) to 7 (strongly agree). Higher scores indicate that emotional regulation strategies are used more frequently. In the present study, the Cronbach’s alpha coefficients were 0.89 for reappraisal and 0.80 for suppression.

#### 2.2.5 The mind-wandering questionnaire

The MWQ is a 21-item self-report measure used to assess the frequency with which individuals engage in mind wandering (30). This questionnaire includes three subscales: spontaneous thinking, attention out of control and overall evaluation. Each item is scored on a 5-point Likert scale ranging from 1 (very rarely) to 5 (always). Higher scores indicate that individuals engage in mind wandering more frequently (22). In the current study, the Cronbach’s alpha coefficient was 0.97 for the total scale.

#### 2.2.6 Early adolescent temperament questionnaire

Effortful control was assessed using the early adolescent temperament questionnaire–revised short form (31). In the Chinese version of this measure of effortful control, 16 items pertaining to activation control, attention and inhibitory control were used to measure effortful control (32). Each item is scored on a 6-point Likert scale ranging from 1 (almost always untrue of you) to 6 (almost always true of you). Higher scores represent higher levels of effortful control. In the current study, the Cronbach’s alpha coefficient for the total scale was 0.71.

#### 2.2.7 The chinese version of the connor-davidson resilience scale

The original version of the CD-RISC includes 25 items (3-factor model) and is used to assess positive psychological qualities that contribute to an individual’s ability to adopt to adversity (33). The Chinese version of the CD-RISC relies on the 3-factor model (6), which includes the dimensions of tenacity, strength and optimism. Each item is scored on a 5-point Likert scale ranging from 1 (not true at all) to 5 (true all the time). Higher scores reflect greater resilience. In the current study, the Cronbach’s alpha coefficients for tenacity, strength, optimism and the total scale were 0.93, 0.94, 0.64 and 0.96, respectively.

#### 2.2.8 The chinese version of the need for uniqueness scale

The original version of the NFU includes 8 items and is used to assess individuals’ positive striving to differentiate themselves from other people (34). The Chinese version of the NFU is a 4-item self-report scale (35). This scale exhibits a single factor structure, and each item scored on a 5-point Likert scale ranging from 1 (not at all/never) to 5 (very much/very often). In the current study, the Cronbach’s alpha coefficient for the total scale was 0.89.

### 2.3 Statistical analysis

First, descriptive statistics and Pearson correlations were analyzed using SPSS 29.0. Subsequently, network analysis was conducted with the assistance of R 4.2.1 software (36). The analysis conducted for this study involved four steps, each of which is described in the following sections.

#### 2.3.1 Network estimation

The MPATS and the relationship network were estimated using the *qgraph* package (version 1.9.2) (37). The qgraph package uses the graphical least absolute shrinkage and selection operator (LASSO) function to produce a Gaussian graphical model. The LASSO function can omit spurious edges to produce a network model corresponding to real connections (38). In the network model, each item is represented by a node, and the associations among nodes are known as edges. The more central the nodes are within the network, the more important they are. A shorter edge between two nodes indicates a higher level of closeness between the nodes in question, and the width and color of the edges represent the strength of association and valence between nodes, respectively. Normally, green or blue lines indicate positive associations, while red lines represent negative associations.

#### 2.3.2 Centrality indices

Two node centrality indices – strength and closeness – were calculated (using Z scored values) with the goal of quantifying the structural importance of each node within the network. The strength of a node is calculated as the sum of the weights of all edges that are directly linked to a node. A node featuring higher strength is exhibits stronger direct connections with many nodes. Closeness reflects the inverse weighted sum of the shortest distance from a node to all other connected nodes. A node that features a higher level of closeness may influence other nodes more quickly. However, certain concerns have been expressed regarding the stability of closeness (39). Therefore, the primary index used in this study was strength.

#### 2.3.3 Network comparison

We used the *Network Comparison Test* (NCT) package to compare the networks obtained using the male and female datasets in terms of invariance of global strength and network structure (40). The NCT is a permutation test in which the difference between the networks associated with two groups is calculated repeatedly for randomly regrouped individuals across three stages. Tests of global strength and network structure were performed, thus generating a *p* value (in which context *p* < 0.05 indicates that the network structure differs significantly between the two samples).

#### 2.3.4 Network robustness

We used the *bootnet* package (version 1.5) to test the robustness of the network structures (40). First, the bootstrapped confidence interval (CI) of each edge was estimated to test the accuracy of the edge weights. Second, the stability of centrality indices was examined by reference to subsamples featuring decreasing sample sizes. The correlation stability (CS) coefficient was also calculated to test the stability of the centrality indices. Finally, bootstrapped difference tests were used to measure the differences in node centralities.

## 3 Results

### 3.1 Preliminary analysis

**Table 2** presents the means and standard deviations (SDs) for each item and dimension of the MPATS, as well as the gender differences in the dimension and total scores. The significance level α was corrected using the Bonferroni procedure. The mean scores for all dimensions exhibited significances between male and female participants. The overall level of mobile phone addiction observed in the female sample was generally higher than that observed in the male sample.

**Table 2:** Results of a Descriptive analysis of the items included in the MPATS-16.

Item/dimension	Male		Female		Gender differences		
	M	SD	M	SD	*t*	*p*	*d*
Withdrawal Symptoms	13.59	4.96	17.65	4.95	-12.88	<0.01	-0.82
1. If I have not used my phone for some time, I immediately check if I have missed any WeChat messages or calls	3.21	1.16	3.84	1.03			
4. I would feel bad if I did not use my phone for a long time	2.35	1.10	3.06	1.08			
6. I would feel lonely without my mobile phone	2.03	1.06	2.85	1.12			
8. If my phone does not ring for some time, I feel uncomfortable and subconsciously check whether I have missed any calls or WeChat messages	1.94	1.03	2.53	1.14			
10. More phone calls or WeChat would make my life more fulfilling	2.05	1.02	2.53	1.07			
12. My phone is a part of me, and when I use it less, I feel as if I have lost something	2.01	1.08	2.84	1.20			
Social Comfort	6.11	2.48	7.85	2.69	-10.29	<0.01	-0.67
2. I would rather chat using my phone than engage in face-to-face communication	1.96	0.92	2.50	1.08			
7. I feel more confident when I communicate with others using my phone	2.17	1.02	2.76	1.03			
16. I feel more comfortable when I communicate with others via cell phone	1.98	0.98	2.59	1.07			
Mood Changes	5.88	2.51	7.65	2.80	-10.10	<0.01	-0.66
3. When waiting for someone, I always ask them where they are using my phone; otherwise, I will be anxious and unbearable	2.18	1.01	2.61	1.07			
11. I often fear that my phone will shut down automatically	1.83	1.06	2.52	1.23			
14. I become anxious and grumpy when my phone does not connect or have a signal	1.87	1.02	2.51	1.14			
Salience	6.92	3.04	9.00	3.50	-10.45	<0.01	-0.63
5. During class/work, I may not be able to concentrate due to phone calls or WeChat messages	1.90	0.98	2.57	1.10			
9. I often experience the illusion of ‘my phone ringing’ or ‘my phone vibrating’	1.69	0.93	2.12	1.07			
13. Classmates and friends often say that I rely on my phone too much	1.69	0.91	2.19	1.05			
15. In class/at work, I often take the initiative to focus on my mobile phone, which affects the lesson/work	1.63	0.86	2.12	1.06			
Total Scores	32.51	11.74	42.15	12.18	-12.52	<0.01	-0.80

d, Cohen’s d.

### 3.2 Network analysis of mobile phone addiction in the male and female samples

#### 3.2.1 Mobile phone addiction networks

**Figure 1:**
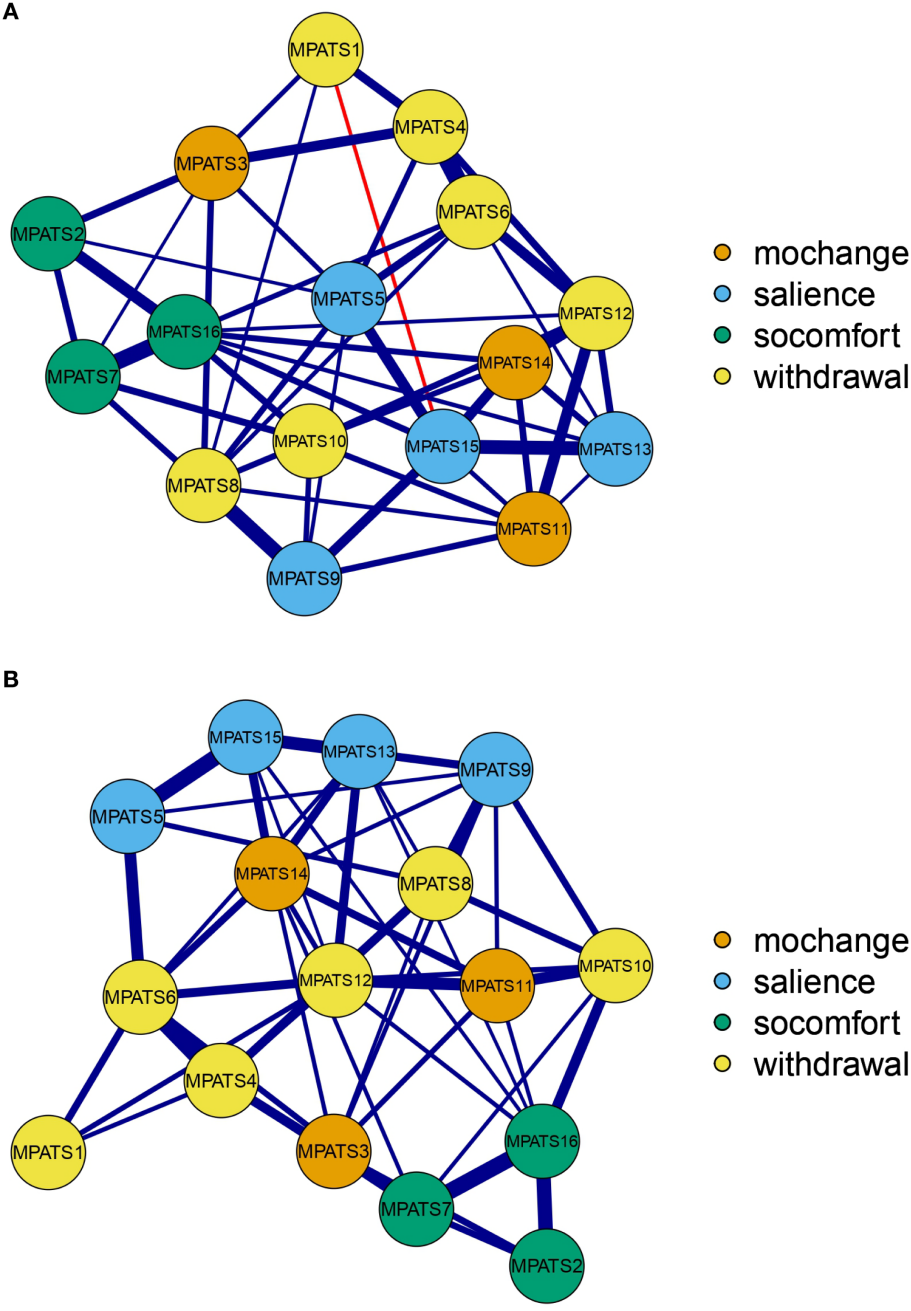
Network structure of mobile phone addiction. **(A)** Male network. **(B)** Female network. *Note.* Mochange, mood changes; socomfort, social comfort; withdrawal, withdrawal symptoms.

**Figure 1** presents the networks pertaining to both the male (**Figure 1A**) and female (**Figure 1B**) samples. With regard to the male sample, the edge between item 4 and item 6 (weight = 0.35) was the strongest among all edges and within the withdrawal symptoms dimension. The edge between item 7 and item 16 (weight = 0.32) was the second strongest within the social comfort dimension. Similarly, with regard to the female sample, the edge between item 4 and item 6 (weight = 0.34) was the strongest among all edges and within the withdrawal symptoms dimension. The edge between item 7 and item 16 (weight = 0.31) was the second strongest among all the edges in this context.

#### 3.2.2 Centrality indices

**Figure 2:**
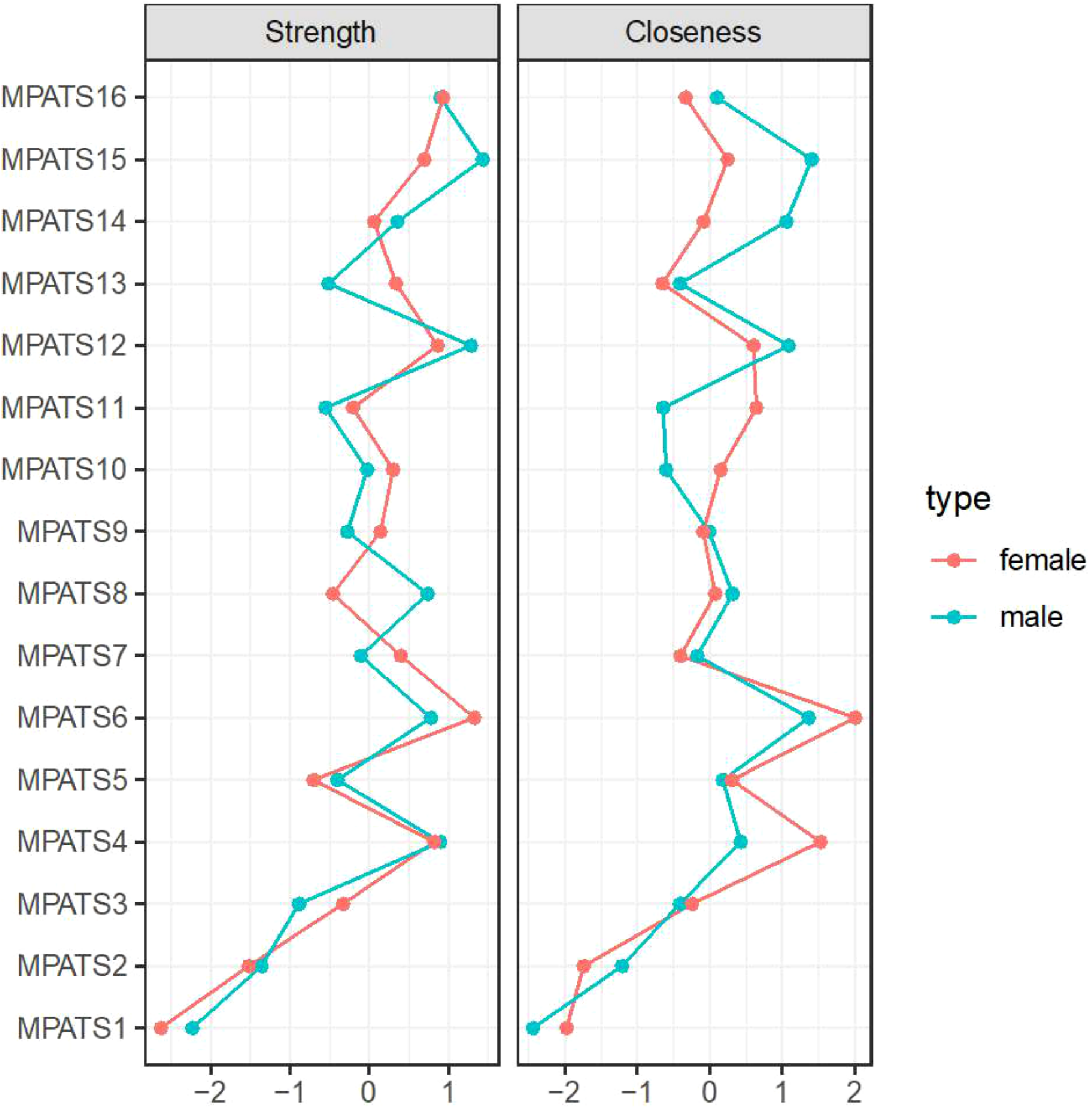
Centrality indices of nodes within the mobile phone addiction networks (z scores).

The centrality indices of the nodes contained in the mobile phone addiction networks are shown in **Figure 2**. With regard to the male sample, item 15 exhibited the highest strength and closeness centrality indices. With respect to the female sample, item 6 exhibited the highest strength and closeness indices.

#### 3.2.3 Statistical network comparison between the two samples

To compare the two networks based on the data collected regarding the two samples, global strength and network structure were compared using the NCT. The results indicated statistically significant differences in global strength (*p* = 0.02) but not in network structure (*p* = 0.52). The global strength value for the male sample was 7.419, whereas for the female sample, this value was 7.042, thus indicating that the male subgroup was associated with stronger connections throughout the whole network than was the female subgroup.

#### 3.2.4 Network robustness

**Figure 3:**
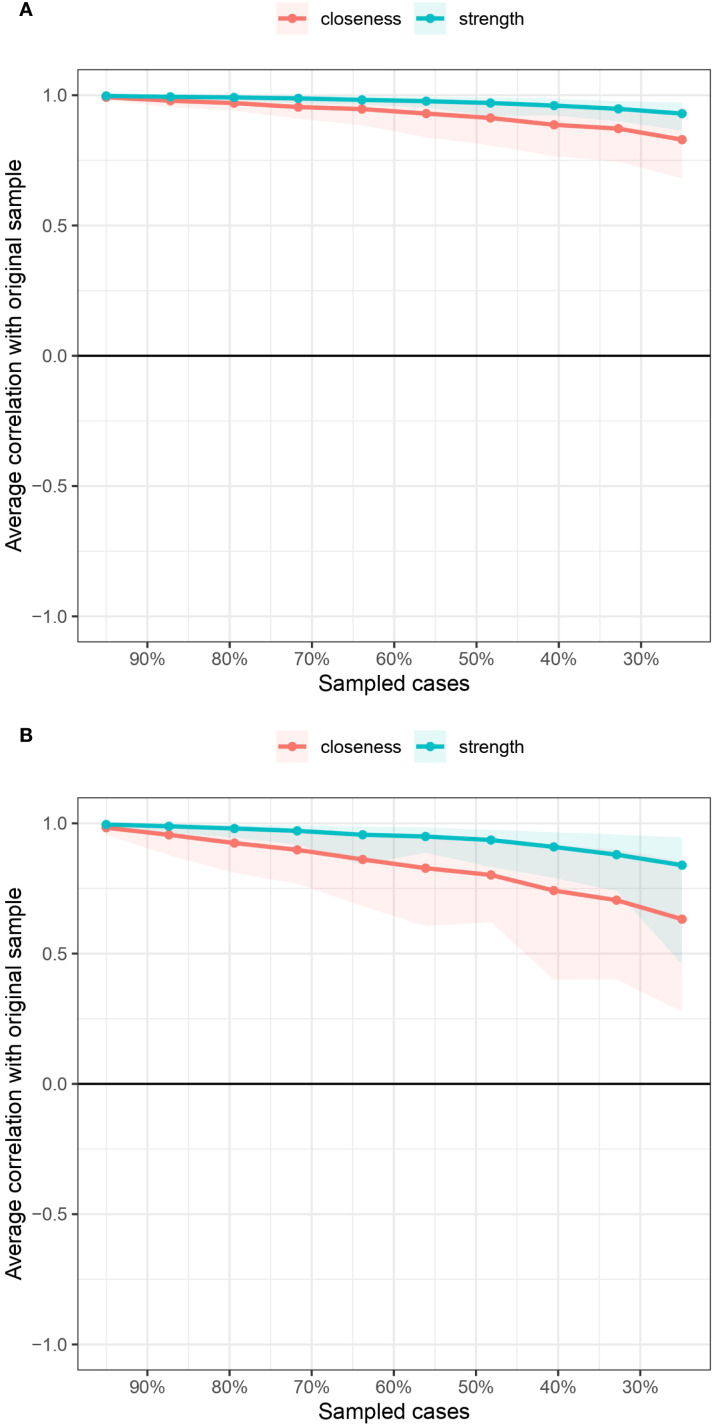
Centrality indices of the mobile phone addiction networks in terms of stability.

The bootstrapped 95% CIs were narrow, thus indicating that the estimated edge weights were accurate and reliable (see **Supplementary Figure 1**). We estimated a bootstrapped sampling distribution (n = 1000) for the networks pertaining to the male and female samples to test the stability of the inferences made regarding the network structures. **Figure 3** presents the stability of the centrality indices associated with the mobile phone addiction networks. With respect to the male and female samples, strength exhibited relatively high stability despite changes in subsample size, while the closeness values exhibited a marginal decrease. In addition, the correlation stability (CS) coefficients were estimated. The CS coefficient should not be less than 0.25 and should preferably be greater than 0.5, which indicates ideal stability. The CS coefficients for strength were good in both networks (0.75 for the male network and 0.671 for the female network). However, the CS coefficient for closeness was not very good for the female network, although it was acceptable (0.672 for the male network, 0.362 for the female network). The bootstrapped difference tests for strength and closeness are shown in **Supplementary Figures 2** and **3**, respectively.

### 3.3 Network analysis of mobile phone addiction and relevant influencing variables

Within network models, each node corresponds to a distinct variable, which represent either a symptom of mobile phone addiction (for example, “feeling lonely without a phone”) or a psychosocial factor (such as “resilience” or “positive reappraisal”). The edges (lines connecting the nodes) indicate the strength of the association between two variables; specifically, thicker edges denote stronger relationships, implying a greater degree of mutual influence between the connected variables. A central node is characterized by having the most robust or numerous connections, functioning as a hub that significantly influences the overall structure of the network.

#### 3.3.1 Network structure

**Figure 4:**
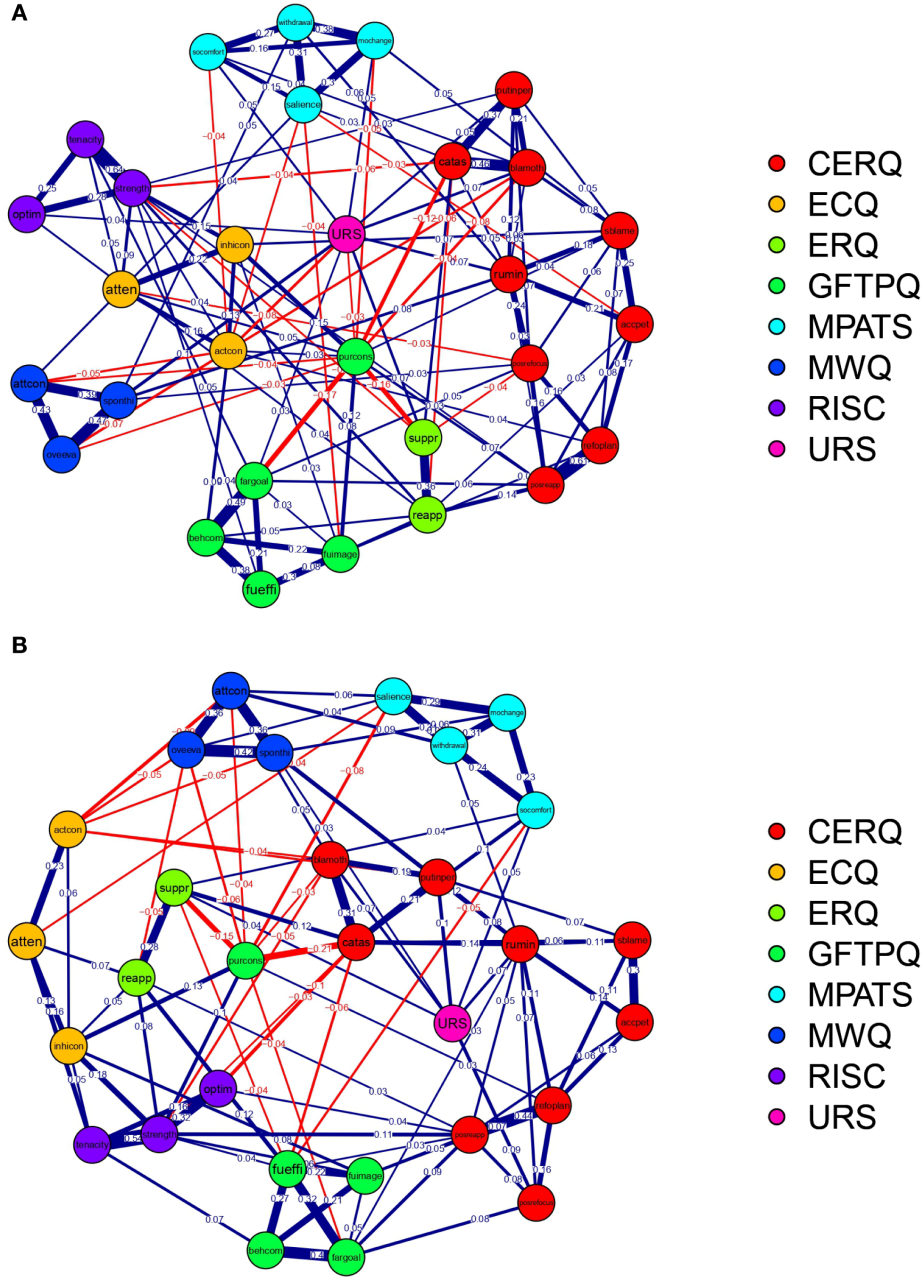
Network structure of mobile phone addiction and relevant influencing variables. **(A)** Male network; **(B)** Female network. CERQ, Cognitive Emotion Regulation Questionnaire; ECQ, Effortful Control Questionnaire; ERQ, Emotion Regulation Questionnaire; GFTPQ, General Future Time Perspective Questionnaire; MPATS, Mobile Phone Addiction Tendency Scale; MWQ, Mind Wandering Questionnaire; RISC, Connor-Davidson Resilience Scale; URS, Unique Requirement Scale.

**Figure 4** presents the network structures for male (**Figure 4A**) and female (**Figure 4B**) participants. A prominent feature observed in both gender-specific networks is the strongest connection within the entire network, linking two components of psychological resilience: “tenacity,” defined as the capacity to persevere through challenging circumstances (e.g., enduring rigorous training), and “strength,” characterized as the ability to grow from adverse experiences (e.g., learning from a lost competition). This association exhibited a high magnitude in males (edge weight = 0.64) and remained substantial in females (edge weight = 0.54). This finding is theoretically coherent, as tenacity and strength function synergistically as a protective dyad that facilitates effective coping mechanisms, thereby explaining their robust interconnection.

#### 3.3.2 Centrality indices

**Figure 5:**
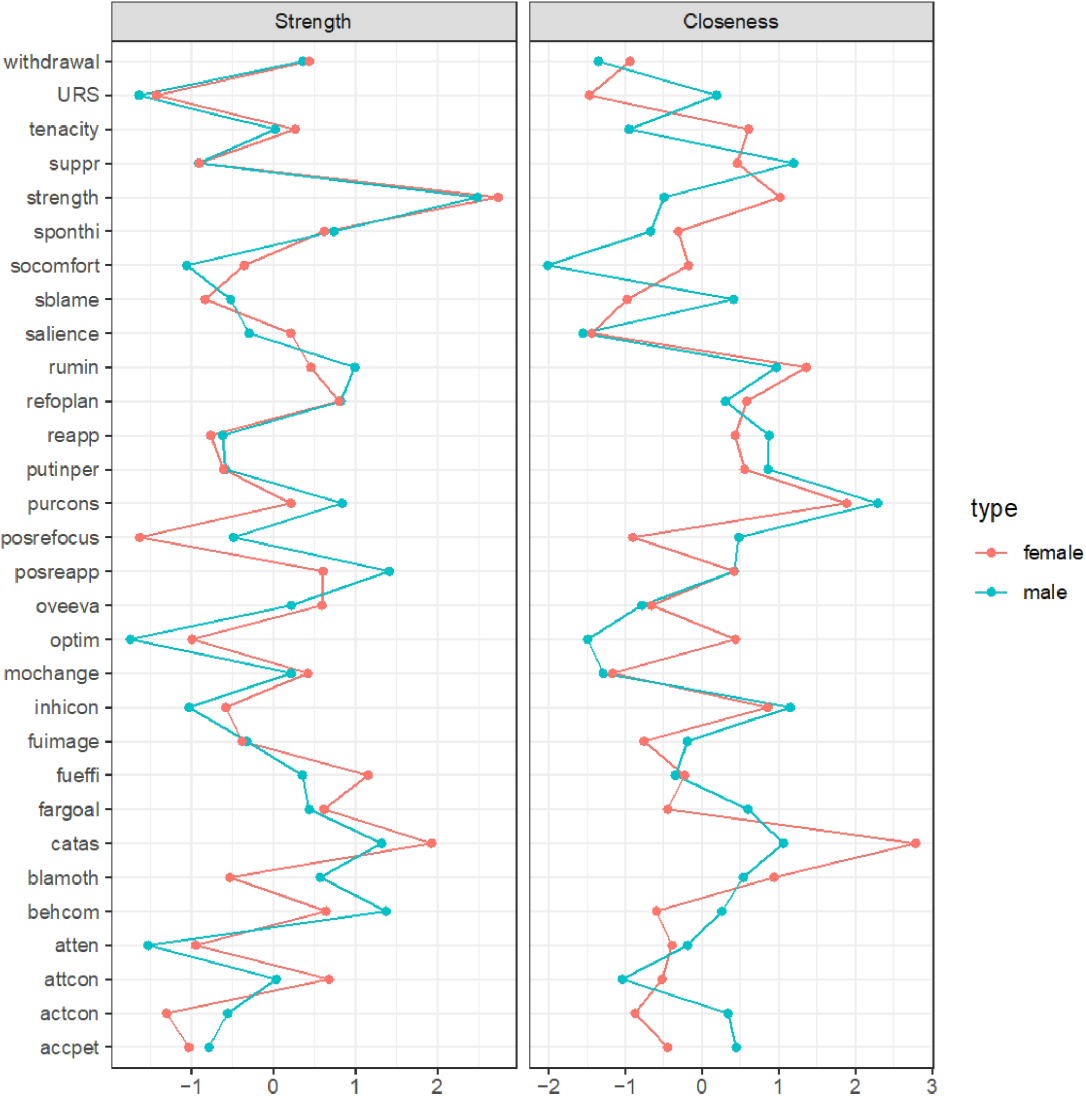
Centrality indices of nodes within the mobile phone addiction and relevant influencing variables networks (z scores).

This study prioritized node strength—defined as the aggregate strength of a node’s connections to other variables—to identify the most influential factors within the network. The centrality indices of nodes within the mobile phone addiction and relevant influencing variables networks (z scores) are shown in **Figure 5**.

For both genders, the most central node corresponded to the “strength” dimension of psychological resilience, as assessed by the Connor-Davidson Resilience Scale (CD-RISC). This indicates that the capacity to recover and grow from setbacks constitutes the single most pivotal variable within the network. It exhibits extensive connections to various addiction symptoms and related factors, including the mitigation of withdrawal symptoms and the reduction of impulsive phone use, thereby playing a critical role in the development of addictive behaviors.

Among males exclusively, the second most central node was identified as “positive reappraisal,” a cognitive strategy involving the reframing of negative experiences. This node functions as a protective hub by attenuating the association between stressors—such as training pressure—and problematic phone use.

Conversely, for females, the second most central node was “catastrophizing,” characterized by excessive worry. This node serves as a risk hub by amplifying the relationship between anxiety—such as pre-competition stress—and addictive phone use, thereby complicating efforts to reduce such behaviors.

#### 3.3.3 Statistical network comparison between the two samples

The Network Comparison Test (NCT) results reveal statistically significant differences in global strength (p = 0.01), whereas no significant differences were observed in network structure (p = 0.22). Specifically, the global strength metric for the male cohort was 15.615, compared to 13.607 for the female cohort, suggesting that the male subgroup exhibited stronger overall connectivity within the network relative to the female subgroup.

### 3.4 Network robustness

**Figure 6:**
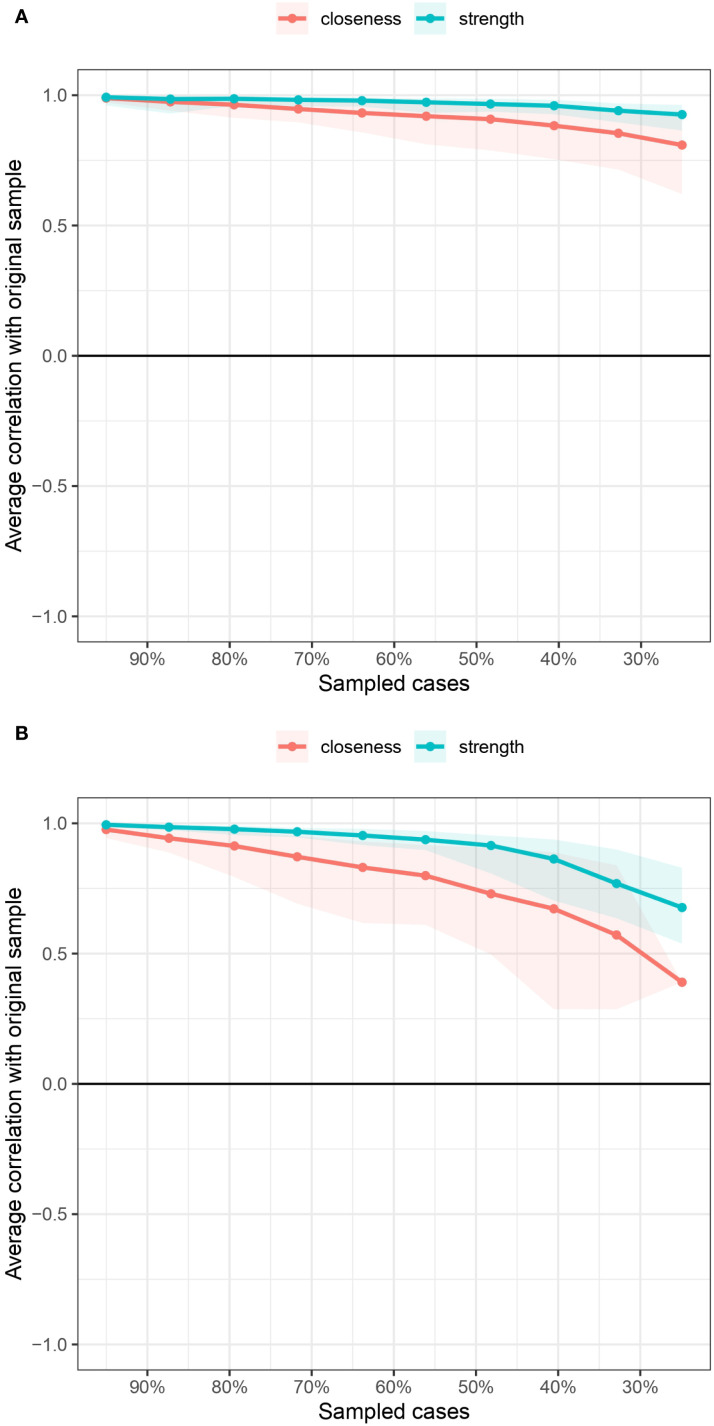
Centrality indices of the mobile phone addiction and relevant influencing variables networks in terms of stability.

The bootstrapped 95% confidence intervals (CIs) were narrow, indicating that the estimated edge weights were both precise and reliable (refer to **Supplementary Figure 4**). **Figure 6** illustrates the stability of centrality indices within the networks. In the male sample, strength demonstrated a relatively high degree of stability despite variations in subsample size, whereas closeness values showed a slight decline. Conversely, in the female sample, both strength and closeness exhibited a pronounced decrease as subsample size changed, with closeness being particularly affected. Furthermore, the correlation stability (CS) coefficient for strength was robust in both networks (0.750 for the male network and 0.672 for the female network). In contrast, the CS coefficient for closeness was acceptable but comparatively lower, especially in the female network (0.595 for the male network and 0.362 for the female network). The results of the bootstrapped difference tests for strength and closeness are presented in **Supplementary Figures 5** and **6**, respectively.

## 4 Discussion

To investigate the specific interrelations among the symptoms of mobile phone addiction and identify risk-increasing and protective factors related to mobile phone addiction, this study examined the network structure of mobile phone addiction as well as the corresponding network structure including relevant influencing factors. The results revealed that the strongest edges were observed within the community in all the networks. This study also identified central nodes that played important roles in these networks. Additionally, we identified gender differences in network characteristics pertaining to both the mobile phone addiction network and the corresponding network including relevant influencing factors.

The results revealed that the specific nodes in the networks of mobile phone addiction were intensively connected in both male and female samples. Item 4 (“I would feel bad if I did not use my phone for a long time”) was closely associated with item 6 (“I would feel lonely without my mobile phone”) for both samples. Both items belong to the withdrawal dimension and reflect negative emotional states arising from restricted phone access, which explains their robust connection. This finding is consistent with previous network studies indicating that the strongest edges often occur within, rather than between, theoretical dimensions (41, 42), underscoring the conceptual and empirical coherence of the withdrawal construct. Similarly, a strong edge was identified between item 7 (“I feel more confident when I communicate with others using my phone”) and item 16 (“I feel more comfortable when I communicate with others via cell phone”), both of which load on the social comfort dimension. This relationship is theoretically plausible, as confidence and comfort in social contexts are closely related psychological experiences (43–45). Our network results empirically support that these two aspects are intertwined in the context of mobile phone use: feeling comfortable in phone-mediated communication may enhance self-confidence, and vice versa, forming a reinforcing cycle that may contribute to the development and maintenance of problematic use patterns. This reciprocity highlights the affective reinforcement mechanisms underlying this dimension of phone addiction.

The networks integrating mobile phone addiction and psychological factors revealed a consistently strong edge between the psychological resilience subcomponents—tenacity and strength—in both genders. This robust connection is conceptually intuitive, as both attributes reflect closely related aspects of resilience: the capacity to persist through challenges (tenacity) and the ability to not only recover from setbacks but also achieve growth and development (strength). This finding aligns with network theory and prior research (35, 46, 47), where the strongest connections typically occur within, not between, conceptual domains. It also resonates with prior work that similarly identified tenacity and strength as strongly connected nodes in a network involving emotion regulation, affect, and resilience (48). What makes this replication noteworthy, however, is its emergence in the specific context of mobile phone addiction. The tight linkage suggests that tenacity and strength function as an interdependent protective unit, potentially enhancing an individual’s capacity to regulate behavior and resist compulsive phone use. The recurrence of this pattern in our study underscores the structural coherence of psychological resilience and highlights its potential role as a buffer in the context of behavioral addiction.

To identify the most influential nodes, we calculated centrality indices, prioritizing the strength metric due to the relatively low stability of closeness estimates—a common finding in network psychometrics (49–51). Interestingly, distinct central nodes emerged across genders in the mobile phone addiction network: for males, the most central symptom was item 15 (“In class/at work, I often take the initiative to focus on my mobile phone, which affects the lesson/work”), whereas for females, it was item 6 (“I would feel lonely without my mobile phone”). The prominence of item 15 among males aligns with prior work highlighting impaired focus as a core feature of mobile phone addiction (52). However, this result contrasts with studies emphasizing loss of control and continued overuse as central (53)—a discrepancy that may stem from cultural or methodological differences in sampling and measurement. For females, the centrality of loneliness without the phone reinforces earlier findings that withdrawal symptoms, particularly affective ones, play a critical role in sustaining problematic smartphone use (52). This divergent pattern invites a meaningful interpretation rooted in potential gender-specific drivers of use. The male network may be primarily driven by a behavioral impulse—a difficulty inhibiting phone use even in situations demanding focus. In contrast, the female network appears centrally organized around an affective state—the fear of isolation and the phone’s role as a social lifeline.

With respect to the network including mobile phone addiction and relevant influencing factors, this study revealed that the strength dimension of psychological resilience was the central node for both males and females. This is a noteworthy finding, as it suggests that strength serves as a common protective factor against addictive phone use across genders. This aligns with previous studies strength buffers against mental health problems such as depression and anxiety (54, 55). This finding is also consistent with a previous network analysis that focused on problematic smartphone use and relevant factors, which reported that resilience was a central node (56). Many studies have demonstrated that psychological resilience has protective effects with regard to mobile phone addiction in either a direct or indirect manner (57–60). What makes this insight particularly compelling is the specific role played by strength. We speculate that strength—as a subcomponent of resilience—equips individuals to adapt to adversity and buffer risk factors of mobile phone addiction such as academic burnout or fear of missing out (57, 61, 62), thereby reducing reliance on phones for emotional regulation or escape. By highlighting strength of resilience specifically, our study clarifies a key malleable target for interventions aimed at fostering healthier digital behaviors.

Additionally, our analysis revealed a striking gender-specific divergence in the cognitive emotion regulation strategies: positive reappraisal served as the central node for males, while catastrophizing was central for females. For males, the prominence of positive reappraisal—a strategy oriented toward reframing negative experiences in constructive terms (63)—aligns with its well-established role as a protective factor for mental health (64–69). Its close functional relationship with resilience (70), which also emerged as highly central in our networks, suggests a reinforcing cycle whereby adaptive coping supports emotional stability and may reduce reliance on mobile devices for mood regulation. In contrast, for females, the centrality of catastrophizing—a pattern of exaggerated negative interpretation—highlights a vulnerability pathway. This finding is consistent with prior work linking catastrophizing to alexithymia and other mental disorders (66, 71–74), particularly in predominantly female samples^74^. We propose that a tendency toward catastrophizing may lead individuals to seek immediate emotional relief through mobile phone use. Furthermore, this process is self-reinforcing: the individual consequently engages in mobile phone use whenever that individual experiences the negative emotional state induced by catastrophizing, resulting in addictive use. This pattern underscores the value of tailoring interventions to gender-specific cognitive-emotional profiles—promoting cognitive reappraisal skills in males and targeting catastrophic thinking in females.

The NCT analysis revealed a notable gender difference in network structure. Despite females reporting higher average levels of mobile phone addiction, the male network exhibited significantly greater global strength. This suggests that while women may have more symptoms, the interactions between these symptoms are more tightly coupled in men. This finding appears to contrast with earlier studies suggesting that stronger network connectivity is associated with greater symptom severity (40, 75), highlighting a potential gender-specific patterning in how mobile phone addiction manifests. One possible interpretation is that for males, addictive phone use may be driven by a more self-reinforcing and interdependent set of behaviors, whereas for females, higher scores may reflect broader—but less reciprocally activated—patterns of use. Although previous studies have reported no gender differences in global strength in networks involving mobile phone addiction and anxiety or internet addiction and depression (76, 77), our results underscore the need to look beyond mean scores and consider structural differences in psychological networks. Future research should further explore what drives these structural divergences and whether they reflect differing motivational or behavioral pathways into mobile phone addiction across genders.

The findings of this study have important implications. Theoretically, by mapping the interactions among specific symptoms, we move beyond aggregate scores to reveal how certain symptoms may activate others—offering a more dynamic understanding of how mobile phone addiction develops and sustains itself. Clinically, the identification of central nodes provides actionable targets for intervention. In line with network theory (78–80), focusing on these highly influential symptoms or factors may produce cascading benefits across the entire symptom network. Crucially, our findings underscore the necessity of gender-sensitive interventions. For males, strategies should prioritize reducing distraction in goal-directed settings (item 15: “In class/at work…”) while bolstering resilience and the use of positive reappraisal. For females, interventions may be more effective if they address emotional dependence (item 6: “I would feel lonely…”), mitigate tendencies toward catastrophizing, and simultaneously strengthen psychological resilience. These tailored approaches acknowledge that the pathways into—and out of—problematic phone use are gendered, and thus demand nuanced clinical responses.

Despite the contributions of our study, several limitations must be considered. First, the cross-sectional design precludes causal inference; longitudinal or experimental studies are needed to examine how symptom networks evolve or respond to intervention over time. Second, we collected data using self-report scales, which may have been subject to recall bias. Future work would benefit from incorporating objective measures of phone use or behavioral assessments. Third, although we included several key factors, other relevant variables such as personality traits and intolerance of uncertainty were not considered^87-88^; their inclusion in future network analyses may yield a more comprehensive understanding. Fourth, although central nodes suggest potential intervention targets, their clinical efficacy must be tested through rigorous experimental or longitudinal designs. Fifth, the use of a convenience sampling and moderate sample size may limit generalizability; future replication in larger and more diverse populations is necessary to enhance the robustness and applicability of our findings. Another notable limitation lies in the pronounced gender imbalance in the sample, which is an inherent outcome of the study’s sampling context—all participants were recruited from military academies. As a structural feature of military academies in China, the student and staff population is predominantly male, and our convenience sampling (designed to access individuals with regular mobile phone use within this specific institutional setting) naturally reflected this inherent gender distribution: of the 1,684 participants, 1,383 (82.1%) were male and 301 (17.9%) were female. This imbalance was not a deliberate design choice but a practical constraint tied to the study’s focus on military academy populations. This gender imbalance may impact the robustness and interpretation of our findings in two key ways. First, it weakens the statistical power of gender-stratified network analyses for female participants. While the larger male sample enabled reliable detection of even weak but meaningful associations (e.g., the link between “phone use during military training breaks” and “effortful control”), the smaller female sample may lack sufficient power to capture such subtle connectivity patterns—potentially leading to underestimation of gender-specific mechanisms unique to military academy women (e.g., how the pressure of balancing military training with daily life interacts with addiction symptoms). Second, it limits the generalizability of female-specific results: the small female subsample not only prevents broad inferences to the wider group of military academy women but also rules out extrapolation to civilian female populations, as military academy women may face context-specific stressors (e.g., gendered expectations in military training) that differ from those of civilian women. Future research should employ longitudinal designs and include a wider range of psychological and social variables to move beyond superficial comparisons and truly explain why and for whom these gendered pathways to mobile phone addiction emerge.

## 5 Conclusion

This research utilized network analysis to systematically investigate the internal interaction patterns among symptoms of mobile phone addiction, as well as the interrelationships at the dimensional level between mobile phone addiction and psychosocial influencing factors. The findings not only support the applicability of the I-PACE model to mobile phone addiction but also highlight resilience, emotion regulation strategies, and effortful control as pivotal nodes within the addiction network. These results further substantiate the model’s focus on dynamic interactions among personal, affective, cognitive, and executive components. Additionally, the study challenges the traditional single-factor causation paradigm of addiction by demonstrating that mobile phone addiction arises from complex reciprocal interactions between symptoms and psychosocial variables. Ultimately, this investigation elucidates the dynamic network structure of mobile phone addiction and its associated factors, offering gender-specific theoretical insights and practical implications for addressing this significant public health issue.

## Data Availability

The raw data supporting the conclusions of this article will be made available by the authors, without undue reservation.
