# Supplementary material for: New perspectives on mobile phone addiction based on network analysis
Source: Front Psychiatry. 2025 Oct 13;16:1665673. doi: 10.3389/fpsyt.2025.1665673 (PMC12556264; doi:10.3389/fpsyt.2025.1665673)
Supplement: Supplementary file 1 [file SupplementaryFile1.docx]

**Supplementary Materials**

1. Figure 1. Accuracy of edge weights in the mobile phone addiction networks for the male and female
2. Figure 2. Bootstrapped difference test for strength in the mobile phone addiction networks for the male and female
3. Figure 3. Bootstrapped difference test for closeness in the mobile phone addiction networks for the male and female
4. Figure 4. Accuracy of edge weights in the mobile phone addiction and related influencing variables networks for the male and female
5. Figure 5. Bootstrapped difference test for strength in the mobile phone addiction and related influencing variables networks for the male and female
6. Figure 6. Bootstrapped difference test for closeness in the mobile phone addiction and related influencing variables networks for the male and female


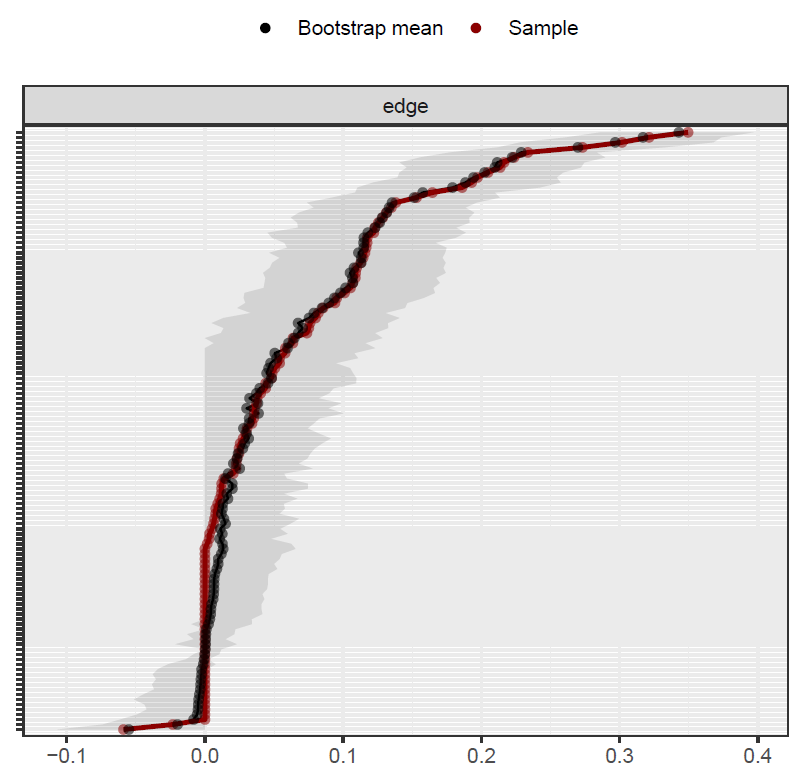

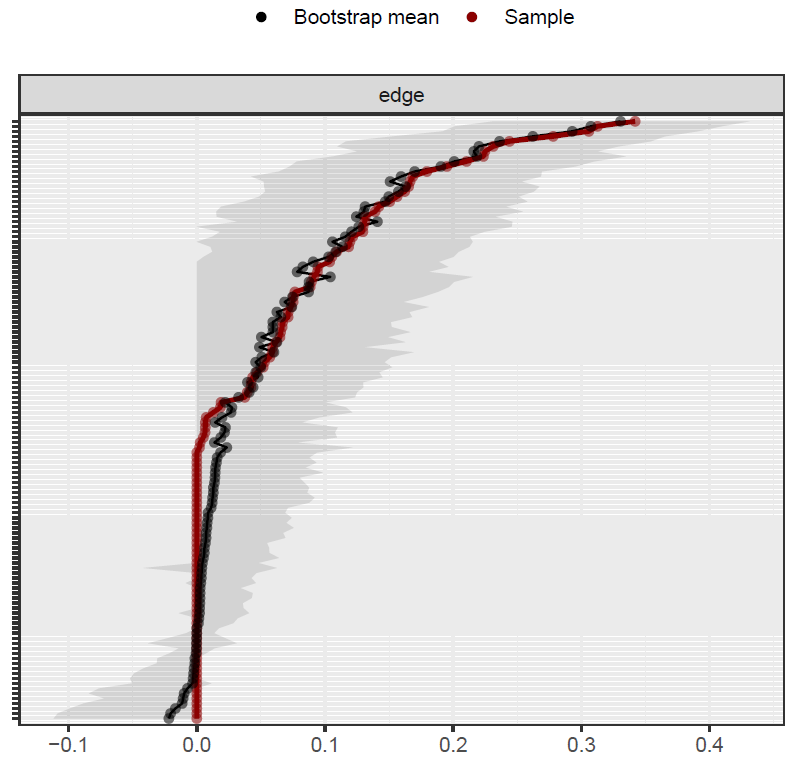


Figure 1. Accuracy of edge weights. Upper panel: male; Lower panel: female.

*Note*: The red line depicts the sample edge weights and the gray bar depicts the bootstrapped confidence interval.


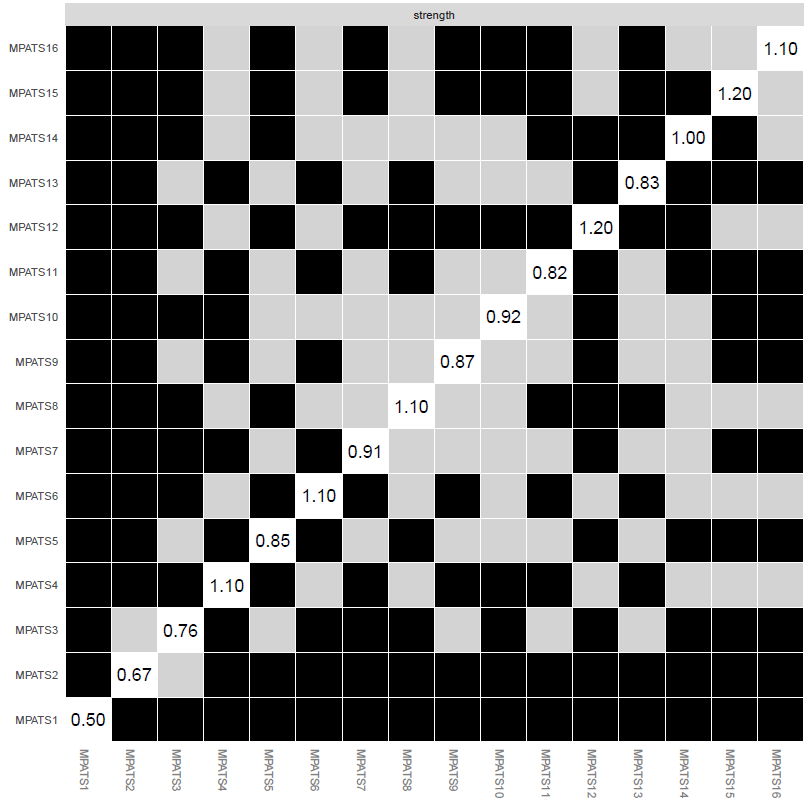


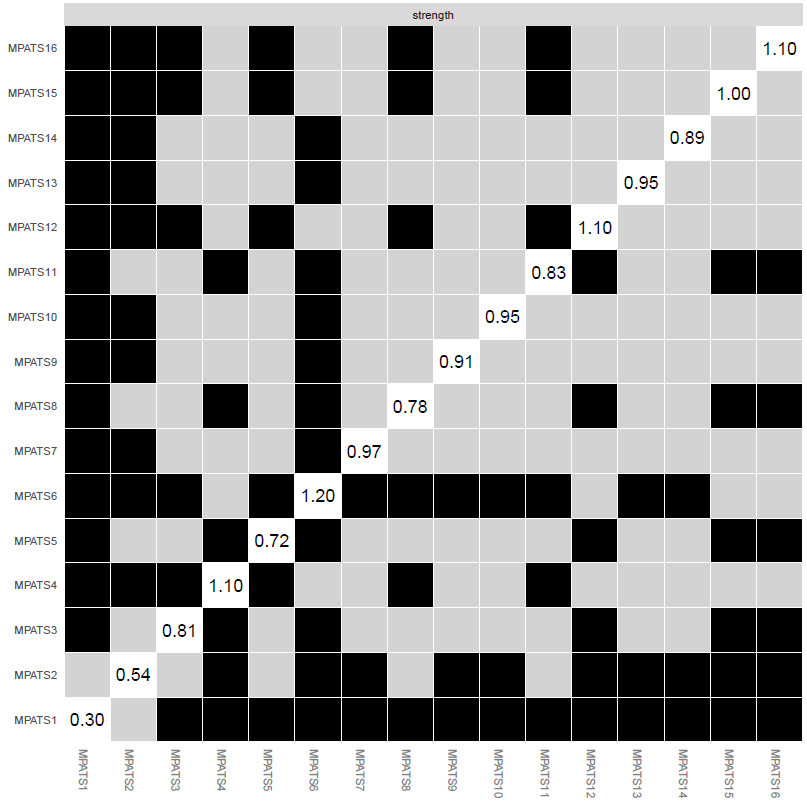


Figure 2. Bootstrapped difference test for strength. Upper panel: male; Lower panel: female.

*Note*: Gray boxes indicate node strength that do not differ significantly from one another, while black boxes indicate node strength that do differ significantly. The number in the white boxes (i.e., diagonal line) represent the value of node strength.


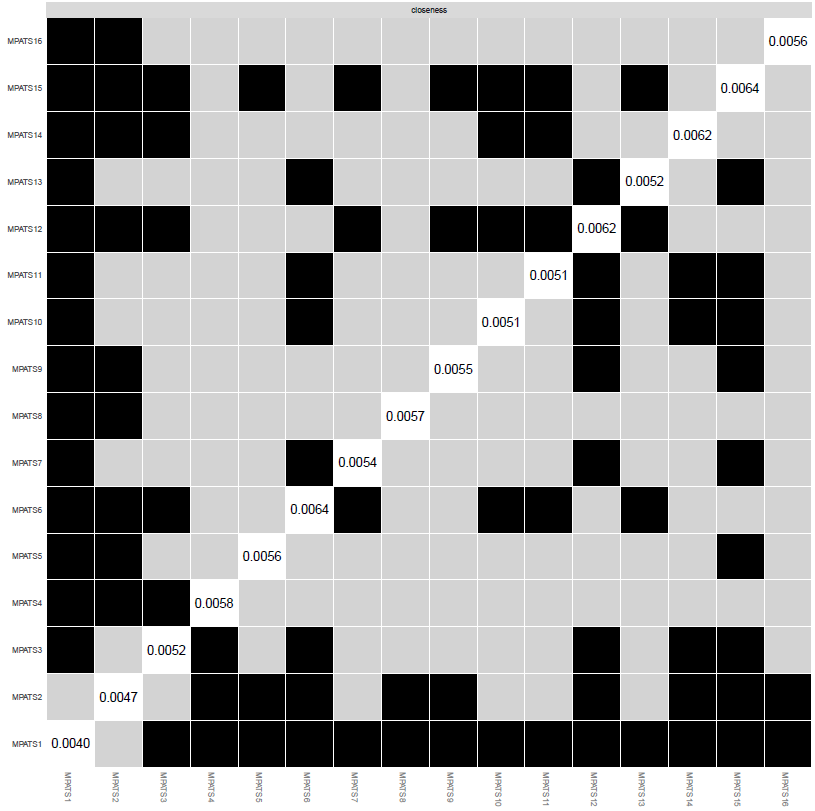

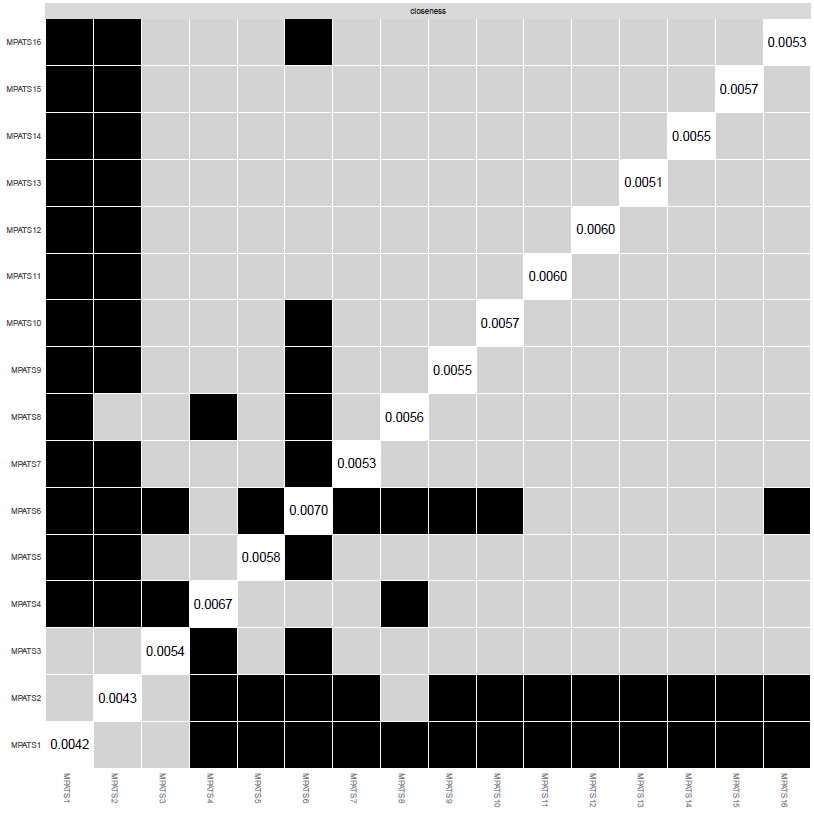


Figure 3. Bootstrapped difference test for closeness. Upper panel: male; Lower panel: female.

*Note*: Gray boxes indicate node closeness that do not differ significantly from one another, while black boxes indicate node closeness that do differ significantly. The number in the white boxes (i.e., diagonal line) represent the value of node closeness.


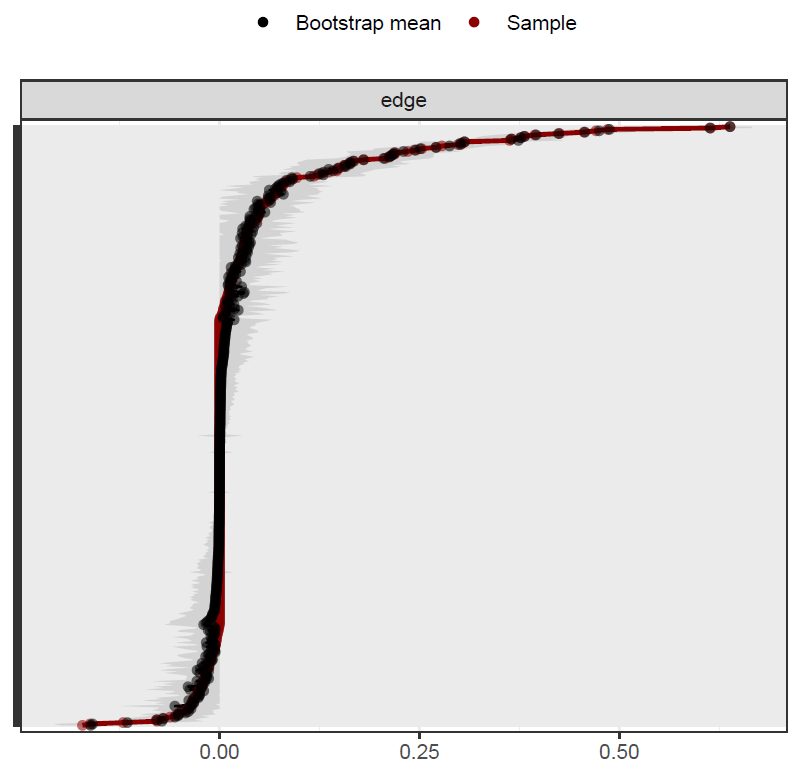


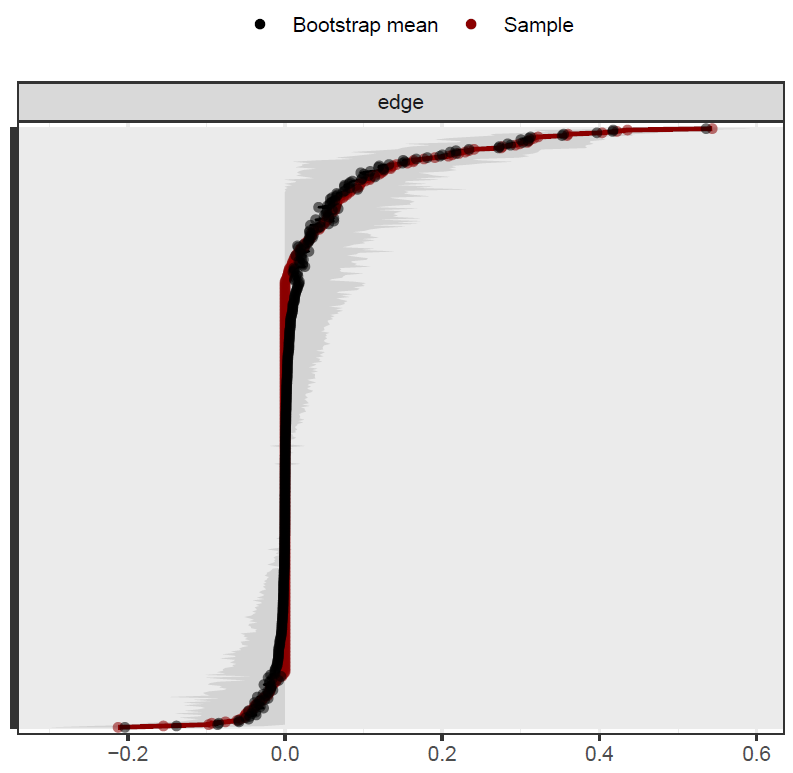


Figure 4. Accuracy of edge weights. Upper panel: male; Lower panel: female.

*Note*: The red line depicts the sample edge weights and the gray bar depicts the bootstrapped confidence interval.


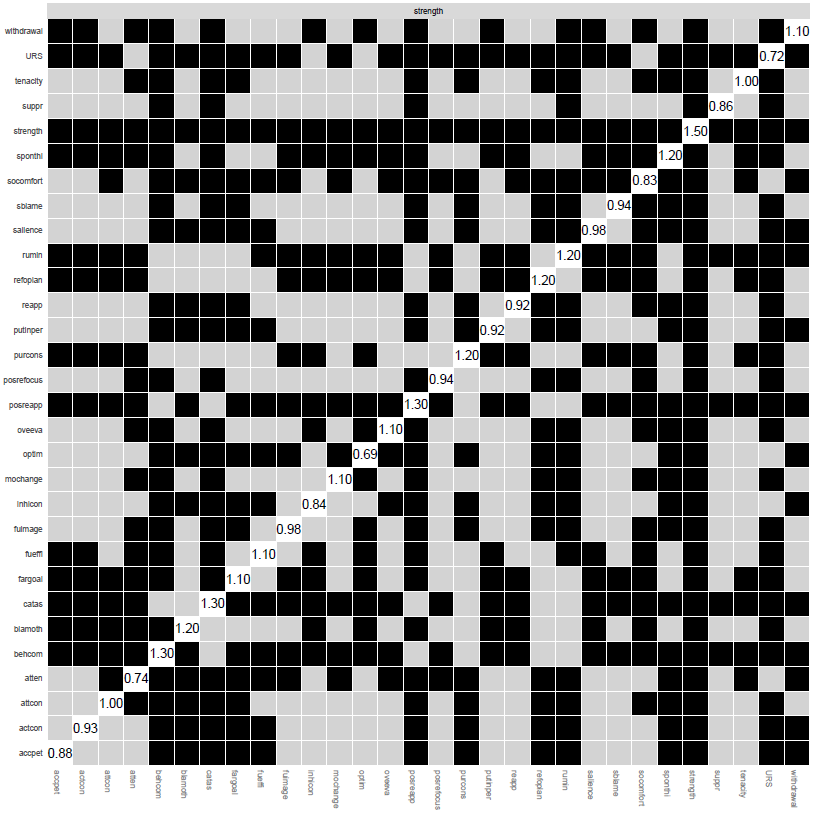


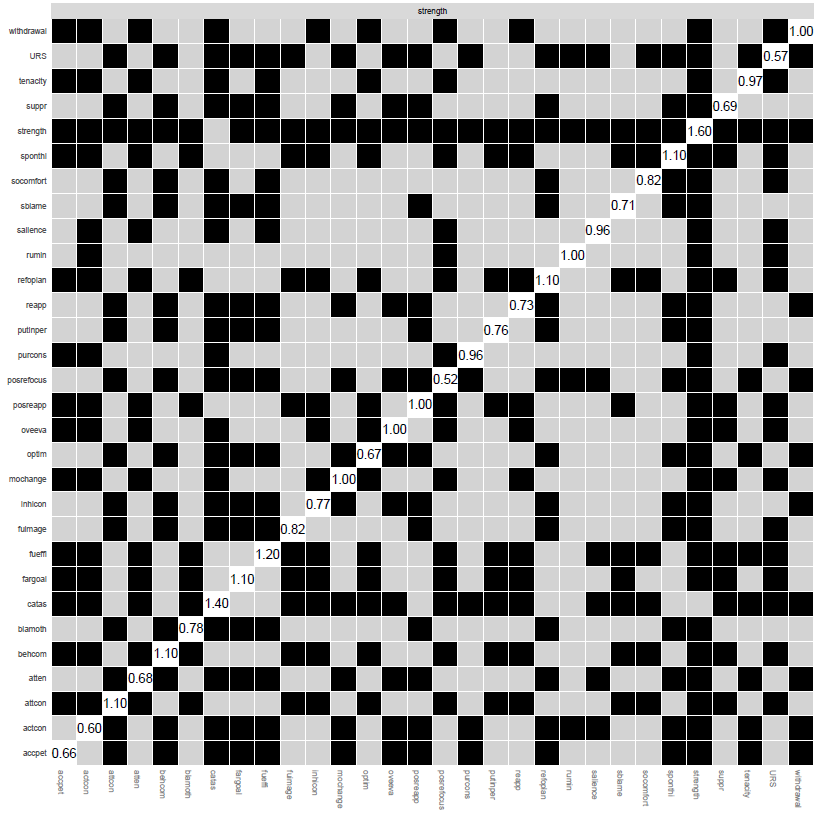


Figure 5. Bootstrapped difference test for strength. Upper panel: male; Lower panel: female.

*Note*: Gray boxes indicate node strength that do not differ significantly from one another, while black boxes indicate node strength that do differ significantly. The number in the white boxes (i.e., diagonal line) represent the value of node strength.


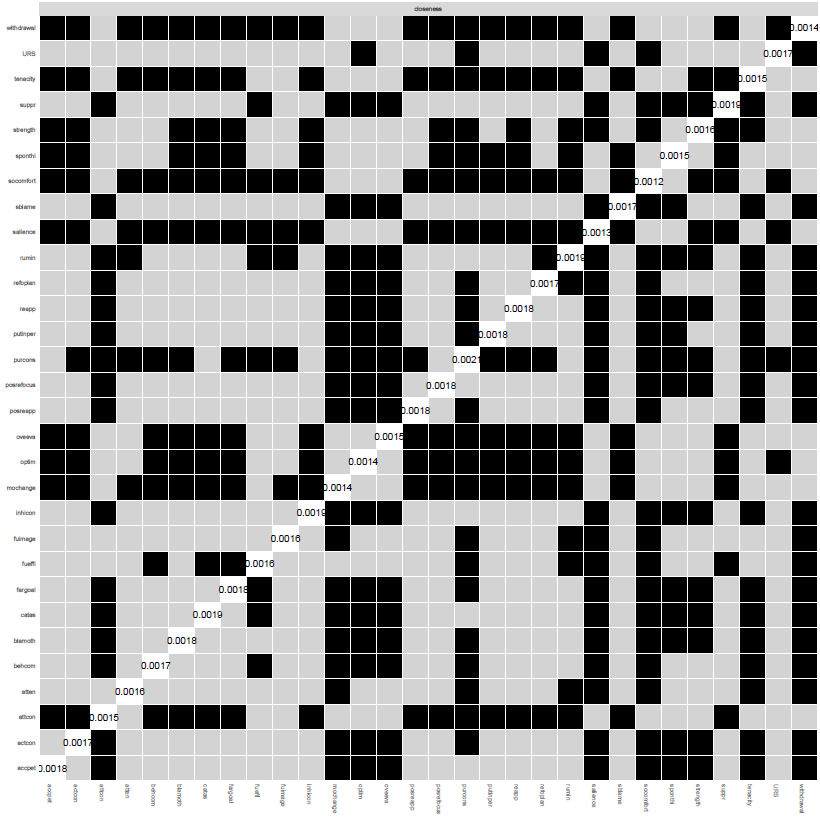


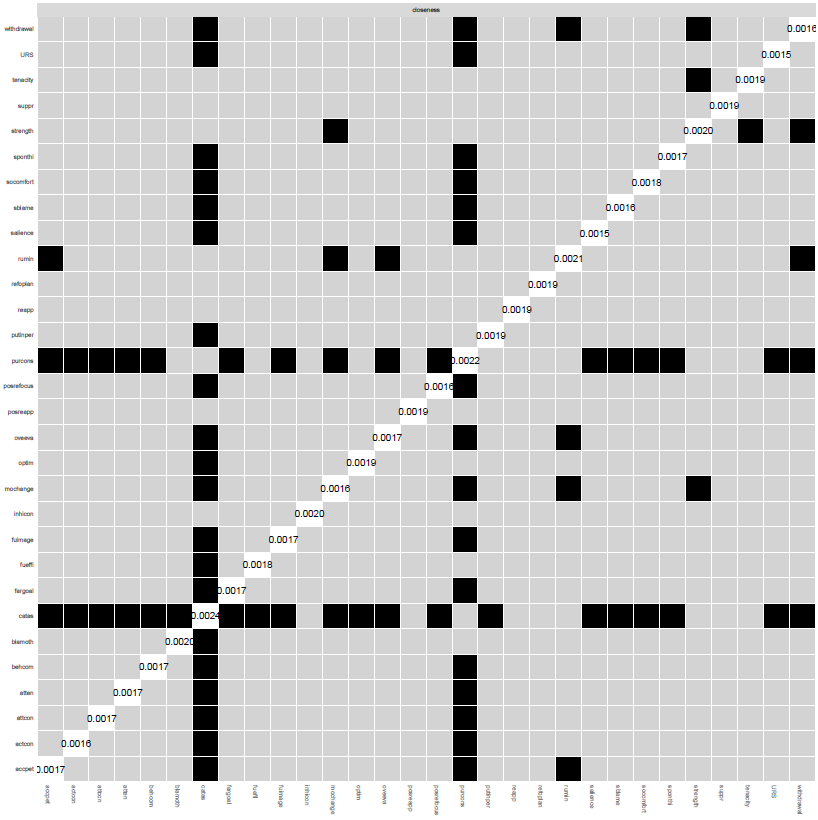


Figure 6. Bootstrapped difference test for closeness. Upper panel: male; Lower panel: female.

*Note*: Gray boxes indicate node closeness that do not differ significantly from one another, while black boxes indicate node closeness that do differ significantly. The number in the white boxes (i.e., diagonal line) represent the value of node closeness.
